# Video-Tachometer Methodology for Wind Turbine Rotor Speed Measurement

**DOI:** 10.3390/s20247314

**Published:** 2020-12-19

**Authors:** Francesco Natili, Francesco Castellani, Davide Astolfi, Matteo Becchetti

**Affiliations:** Department of Engineering, University of Perugia, Via G. Duranti 93, 06125 Perugia, Italy; francesco.natili@studenti.unipg.it (F.N.); davide.astolfi@studenti.unipg.it (D.A.); matteobecchetti@alice.it (M.B.)

**Keywords:** video tachometer, image processing, vision based measurement, wind turbines, non-stationary machines, correlation matrix

## Abstract

The measurement of the rotational speed of rotating machinery is typically performed based on mechanical adherence; for example, in encoders. Nevertheless, it can be of interest in various types of applications to develop contactless vision-based methodologies to measure the speed of rotating machinery. In particular, contactless rotor speed measurement methods have several potential applications for wind turbine technology, in the context of non-intrusive condition monitoring approaches. The present study is devoted exactly to this problem: a ground level video-tachometer measurement technique and an image analysis algorithm for wind turbine rotor speed estimation are proposed. The methodology is based on the comparison between a reference frame and each frame of the video through the covariance matrix: a covariance time series is thus obtained, from which the rotational speed is estimated by passing to the frequency domain through the spectrogram. This procedure guarantees the robustness of the rotational speed estimation, despite the intrinsic non-stationarity of the system and the possible signal disturbances. The method is tested and discussed based on two experimental environments with different characteristics: the former is a small wind turbine model (with a 0.45 m rotor diameter) in the wind tunnel facility of the University of Perugia, whose critical aspect is the high rotational speed (up to the order of 1500 RPM). The latter test case is a wind turbine with a 44 m rotor diameter which is part of an industrial wind farm: in this case, the critical point regards the fact that measurements are acquired in uncontrolled conditions. It is shown that the method is robust enough to overcome the critical aspects of both test cases and to provide reliable rotational speed estimates.

## 1. Introduction

The condition monitoring of non-stationary rotating machines [1,2] is a complex task in general, and it is very important due to the wide industrial applications, in particular as regards renewable energy systems [3]. For wind energy technology, the maintenance costs are crucially important and have to be taken into account in the economic balance of industrial plants [4,5,6]. A wind turbine failure does not reflect only a lower availability of the power plant or a loss of production, but due to the increasing size of industrial wind turbines [7,8], even organizing and carrying out repair activity at high levels or offshore [9,10,11] can be extremely expensive. Furthermore, it should be noted that most modern wind turbines transform the slow rotation of the shaft into the fast rotation of a generator through a gearbox: this implies the necessity of monitoring gears and bearings [12,13] and recognizing faulty signatures with respect to gear mesh frequencies in non-stationary operation.

For non-stationary rotating machines in general and for wind turbines in particular, it is therefore crucial to reliably and precisely measure the rotational speed in order to detect future faults with appropriate advance notice. Continuous condition monitoring systems are typically based on the use of encoders or resolvers to measure the rotational speed based on the mechanical contact with the rotating element. Instead, it might be of practical interest to formulate methodologies that are not based on intrusion or contact with the machine itself.

This kind of approach can be particularly useful for spot analysis in industrial systems, as for example in wind farms. In fact, an important issue in industrial sectors characterized by the use of complex systems regards data openness and site accessibility: this often happens, for example, in the wind energy industry, where wind turbine owners and constructors are typically different subjects. As, for example, the operation of wind farms and the access to wind turbines is in general regulated by agreements between owners and technological providers, the possibility of analyzing certain aspects of a wind turbine’s dynamical behavior could likely result in the capability of monitoring it reliably, without intruding and possibly without requiring too much information (as, for example, the gearbox geometry and therefore the gear meshing frequencies).

Based on these considerations, previous work by the authors of this study has been devoted to an innovative approach to wind turbine drive-train condition monitoring [14]: the general concept of that study was to collect appropriate vibration measurements at wind turbines in order to circumvent the drawbacks associated with this kind of data source (for example, the proprietary nature of data recorded by industrial turbine condition monitoring systems). The proposal put forward in [14] was to measure vibrations non-intrusively at the tower of the wind turbines at human height. The rationale for this choice is the avoidance of the interruption of the normal operation of the machine and the exclusion of every possible safety issue associated with human intervention in an industrial wind farm. This idea introduces novel drawbacks, because in general the transfer function from the target drive-train to the tower is not known: nevertheless, in [14], the method has been shown to be effective for the diagnosis, with months of advance notice, of bearing faults in an industrial wind farm. Further development of the analysis proposed in [14] shows the necessity of measuring the rotor speed non-intrusively, with precision and adequate sampling time. The present study deals exactly with this issue.

Therefore, the objective of this study is to formulate and test a contactless methodology for the measurement of wind turbine rotor speed from video acquisitions at ground level. There are several possible applications of this kind of approach in the wind energy industry and research: the above discussion indicates that it could complete the drive-train condition monitoring technique for industrial wind farms, as proposed in [14]. Such methods could also be applied in the context of condition monitoring for refurbished wind turbines, which are typically not (and presumably will not be) equipped with condition monitoring systems. Furthermore, interesting applications can be conceived in the context of small wind energy systems.

Few studies in the literature have been devoted to this kind of issue in the context of wind energy, despite the rising trend of vision-based measurement in engineering (as stated in [15]). To the best of the authors’ knowledge, a similar methodology is proposed in [16], in which the approach is tested on a permanent magnet synchronous motor; in that study, it is indicated that a valuable application would be non-stationary rotating machines, such as wind turbines. It should be noted that the present study is more general compared to that in [16] in terms of image analysis and signal processing. In [16], a portion of the target image is selected by the users for rotational speed reconstruction, while in the present study, the entire frame captured by the camera is employed; therefore, the method of this study is more easily automatable. Furthermore, in [16] the rotational speed is estimated from the analysis of the average of the Fourier transform, while in this study, the spectrogram has been employed because it has been considered more robust with respect to disturbances in the signal. A recent study dealing with the application of video-tachometer methods to wind turbines is presented in [17]: the approach is based on computer vision, deep learning and video mining. First, a Single Shot Multibox Detector (SSD) is employed to detect the hub of the wind turbine in the image; subsequently, binary classification algorithms are used to discriminate between blade and non-blade areas. This process is repeated for all frames in a specified time window, and it can be used to estimate the angular speed: the method is validated on images and a video (with 30 frames per second) captured at operating wind farms.

An important aspect of the present study is that two different experimental environments are considered and each is characterized by different critical points; thus, the robustness of the measurement and processing methodology is discussed. The considered experimental test cases are as follows:

- The measurement of the rotor speed of a small horizontal-axis wind turbine model (with a 0.45 m rotor diameter) in the wind tunnel facility of the University of Perugia. In this case, the critical points regard the high rotor speed reached by the prototype (hundreds of RPM) and the rapid variability of the rotor speed for this kind of machine [18].
- The measurement of the rotor speed of an industrial wind turbine (with a 44 m rotor diameter) in a wind farm. In this case, the rotor speed is in the order of a few dozens of revolutions per minute, and the critical points are related to the post-processing, because disturbances can be induced by sunlight and varying backgrounds.

The structure of the manuscript is therefore as follows: in Section 2, the method, including the equipment and algorithms, is introduced. The test cases are described in Section 3. In Section 4, results are presented and discussed. Conclusions are drawn and further directions are indicated in Section 5.

## 2. Methods and Facilities

In this section, a description of the mathematical background for the proposed video-tachometer application is presented. Subsequently, the experimental setup of the laboratory and field tests is shown.

### Video-Tachometer Working Principle

The main goal of the proposed contactless video-based tachometer method was to perform an estimation of wind turbine rotor speeds. The general idea was to collect a reference frame in which one of the wind turbine blades was clearly visible and then to record a series of video acquisitions while the rotor was moving. During the post-processing of the data, the reference image was compared with each frame of the video using a correlation algorithm, obtaining a vector with the calculated correlation values in which the maximum value of the correlation occurred each time one of the blades assumed the same position as that in the reference frame. In this way, knowing the camera frame rate, it was possible to construct a virtual tachometer signal showing peaks with a frequency proportional to the rotor’s rotational signal, where the proportionality factor depended on the number of blades.

Mathematically, the comparison between the frames was performed by evaluating the Pearson product–moment correlation coefficient. Images were acquired as 8 bit black and white images, with an m×n dimensional matrix. Subsequently, the matrixes were reshaped in order to obtain a vector, whose dimension was n·m. Labeling as $\hat{X}$ and $\hat{Y}$, respectively, the matrix of the reference image and of a sample frame of the video, both having dimensions m×n equal to the height and width of the selected resolution of the images, Equation (1) can be written as follows:

(1) $$\begin{array}{cc} \hat{X}=\left[\begin{array}{cccc} x_{1,1} & ... & ... & x_{1,n}\\ \; & . & . & \\ \; & . & . & \\ \; & . & . & \\ x_{m,1} & ... & ... & x_{m,n} \end{array}\right] & \hat{Y}=\left[\begin{array}{cccc} y_{1,1} & ... & ... & y_{1,n}\\ \; & . & . & \\ \; & . & . & \\ \; & . & . & \\ y_{m,1} & ... & ... & y_{m,n} \end{array}\right] \end{array}$$

As the frames were acquired in black and white with an 8 bit depth, the elements of the matrice assumed values between 0 and 255.

A further step was to reshape the matrices into a single dimension vector, as shown in Equations (2) and (3):

(2) $$X=\left[\begin{array}{cccc} x_{1}=x_{1,1} & x_{2}=x_{1,2} & ... & x_{mxn}=x_{m,n} \end{array}\right]$$

(3) $$Y=\left[\begin{array}{cccc} y_{1}=y_{1,1} & y_{2}=y_{1,2} & ... & y_{mxn}=y_{m,n} \end{array}\right]$$

After the reshaping, the covariance matrix *C* was calculated according to Equations (4)–(6):

(4) $$C_{1,1}=var\left(X\right)=\frac{1}{nm-1}\sum_{i=1}^{nm}{\left(X_{i}-\bar{X}\right)}^{2}$$

(5) $$C_{2,2}=var\left(Y\right)=\frac{1}{nm-1}\sum_{i=1}^{nm}{\left(Y_{i}-\bar{Y}\right)}^{2}$$

(6) $$C_{1,2}=C_{2,1}=cov\left(X,Y\right)=\frac{1}{nm-1}\sum_{i=1}^{nm}\left(X_{i}-\bar{X}\right)\left(Y_{i}-\bar{Y}\right)$$

where $\bar{X}$ and $\bar{Y}$ are the mean values of *X* and *Y*, respectively.

From the covariance matrix, the Pearson correlation coefficient [19] could be calculated as shown in Equation (7):

(7) $$R=\frac{C_{2,1}}{\sqrt{C_{1,1}C_{2,2}}}.$$

**Figure 1:**
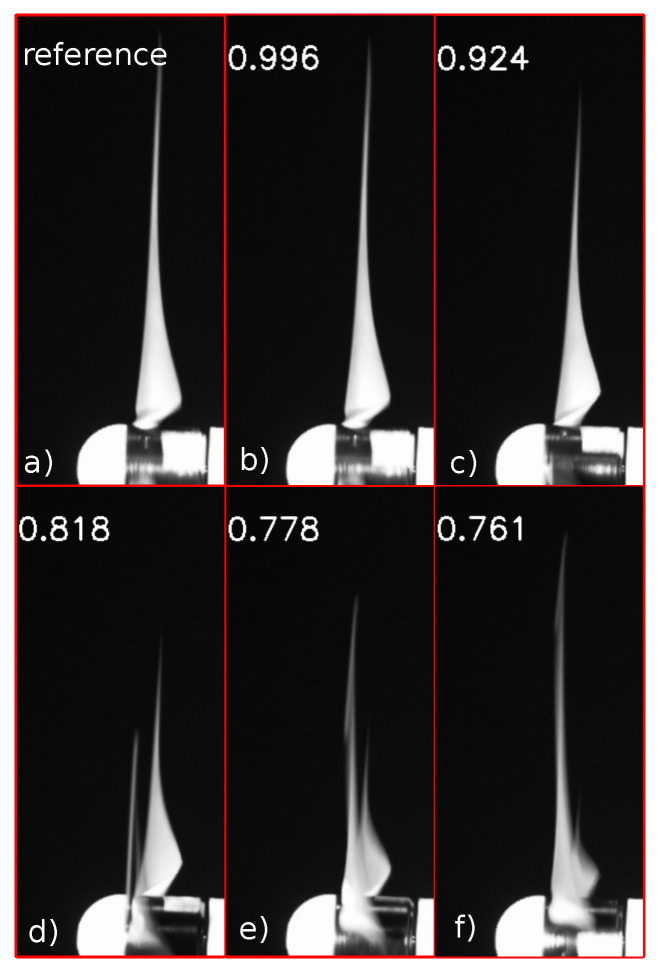
Referenceimage (**a**) and variation of correlation coefficient (**b**–**f**).

In this way, for each frame, a corresponding value of correlation was obtained. The correlation can be considered to be a useful parameter to evaluate the similitude between two objects [20,21], which in this case were the two vectors *X* and *Y*, representing the reference and a frame of the video. This property of the correlation was used in the algorithm to determine when the similitude reached its maximum and extract its periodicity, which was expected to be proportional to the rotational value. The correlation coefficient was close to its maximum value of 1 as the blade assumed a position closer to that of the reference image. The minimum value of the correlation, which could be hypothetically equal to zero, in the practical case was found at about 0.7–0.8, as there were some parts of the pictures that remained unchanged in all the frames (for example, the background and part of the hub). Figure 1 shows how the value of the correlation varied during the revolution of the rotor.

**Figure 2:**
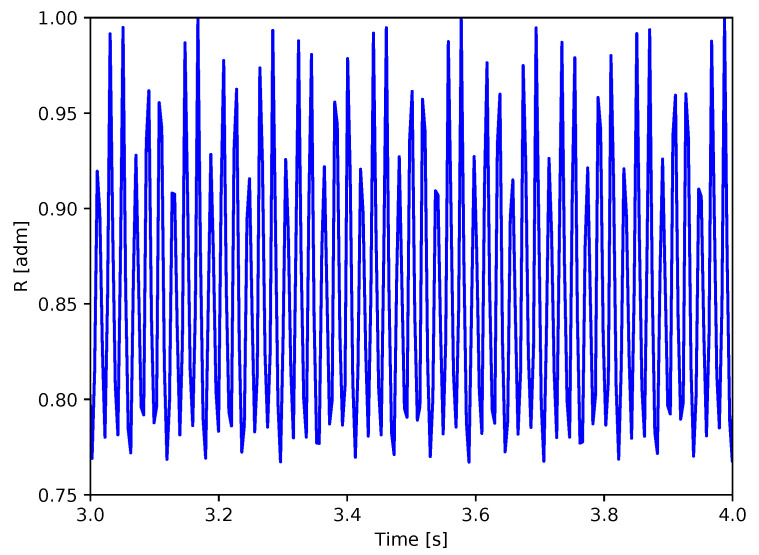
Example of virtual tacho signal.

Figure 2 shows an excerpt of a virtual tachometer signal obtained from the correlation of a video acquisition: it can be seen that, from one revolution to another, the maximum value of the correlation, as well as the minimum value, did not assume a constant value but showed some variability. This effect can be caused by many factors, such as vibrations, blade deflection, a slight eccentricity of the rotor or even from a difference between the azimuth position of the blade in the reference image and in the acquired frame. All these occurrences led to a non-constant value of peaks, introducing a subtle challenge to the determination of the effective rotational speed that should be evaluated as the number of peaks in a given temporal interval. As the maximum (or minimum) value variable, two thresholds were defined as extreme limits above or under which the maximum or minimum were considered to be reached.

This approach, although feasible, is not recommended in this application because it would add an element of uncertainty, given the fact that the calculated speed may give inaccurate results depending on the choice of the threshold values. A more robust approach for the extraction of rotational speed from the virtual tachometer can be obtained using a spectrogram, or rather by dividing the signal into multiple time intervals and calculating the spectrum at each section. In this way, the spectrogram shows a peak corresponding to the blade passing frequency, which is three times the rotational frequency. The spectrogram is formally computed through a Short-Time Fourier Transform (STFT), which in its discrete formulation is given in Equation (8):

(8) $$STFT\left(T,f\right)=\sum_{t=0}^{k-1}z\left(t\right)h\left(t-T\right)e^{\frac{-2\pi itf}{k}},$$

where z(t) is the input signal, h(t) is a Tukey window function, *T* and *f* are the time and frequency indexes, respectively, and *k* is the number of samples used for the Fourier transform.

The spectrogram gives an array whose elements are the square modules of the STFT(T,f) as a result.

**Figure 3:**
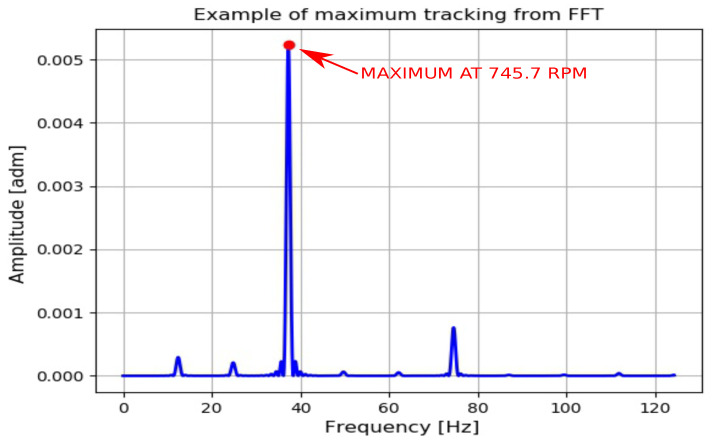
Example of maximum tracking from the Fast Fourier Transform (FFT): the value of 745.7 RPM has been computed by determining the frequency at which the maximum peak appears (blade passing frequency) and dividing by $\frac{no.ofblades}{60}=\frac{3}{60}=0.05$.

**Figure 4:**
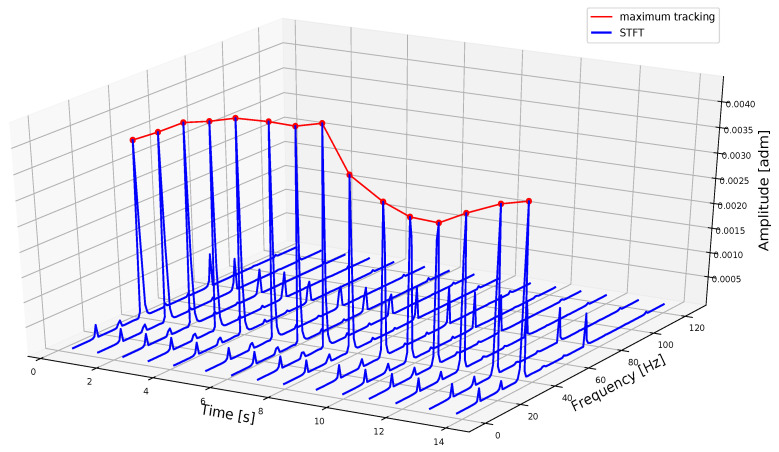
Visualization of maximum tracking with multiple Short-Time Fourier Transforms (STFTs).

If a single FFT that composes the spectrogram is considered, a plot similar to Figure 3 is obtained, where peaks related to different frequencies are present. As expected, the maximum value of each FFT is in correspondence with the blade passing frequency; it is then possible to track all these values to calculate the rotation speed for a machine operating in non-stationary conditions. In Figure 4, the principle of maximum tracking is shown with many FFT splaced side-by-side in the time–frequency domain. The spectrograms in the following sections are 2D representations of Figure 4 with higher resolution in frequency, and the amplitudes are depicted with a color intensity scale. The tracking algorithm may fail if some peaks in FFT are higher than that related to the blade passing frequency: this aspect is discussed in detail in Section 4, after calculating the signal-to-noise ratio (SNR) value and percentage difference.

**Figure 5:**
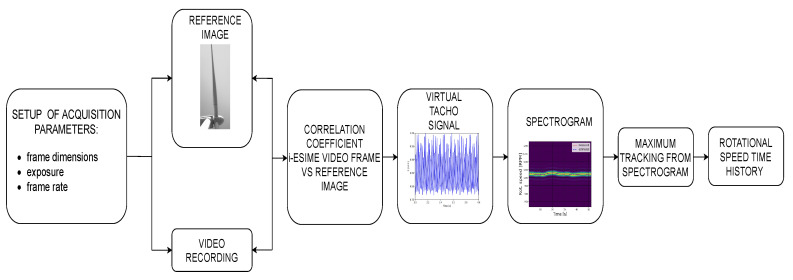
Block diagram of correlation algorithm.

Figure 5 shows all of the steps of the correlation algorithm used to estimate the rotational speed of wind turbines. In Section 4, the method is first validated to ensure its reliability through wind tunnel tests and then applied to a full-sized turbine operating in the field.

## 3. Test Cases and Experimental Set-Up

### 3.1. Wind Tunnel Tests

The correlation-based algorithm for the estimation of rotating speed has been tested in the “R.Balli” wind tunnel facility in the Department of Engineering at the University of Perugia (Italy). The closed-loop tunnel has an open test chamber with a squared inlet and outlet ducts of 2.2×2.2 m and 2.7×2.7 m, respectively. Wind speeds can reach up to 45 m/s thanks to a fan driven by a 375 kW electric motor. More details about the wind tunnel facility can be found, for example, in [18].

A small-scale wind turbine with a 0.45 m rotor diameter has been used for this test: its blades are constructed from titanium and the hub, as well as the nacelle, has been internally designed for research purposes and 3D printed. The rotational speed of the turbine can be controlled by varying the electrical resistance applied to the generator; in this case, the electric load was kept constant for all tests at 3 Ω. To validate the video tachometer method, an optical tachometer was installed on the nacelle of the turbine, and a marker was applied to the rotor in order to obtain a transistor–transistor-logic signal from the sensor, from which the rotational speed was calculated. The aim of the test was to evaluate the capability of the correlation method to estimate the rotational speed, with the measurement from the optical tachometer used as the term of comparison. In the following, the rotor speed obtained from the correlation algorithm is labeled as the “estimated speed”; in contrast, the speed from the sensor is called the “measured speed”. To investigate a comprehensive series of case studies, many test runs were realized with different flow conditions, which were both steady and unsteady. In particular, three tests were conducted with steady flow at 4, 5 and 6 m/s of wind speed; two tests were conducted with sinusoidal wind, with a 4.5 m/s mean value and ±0.3 m/s and ±0.6 m/s amplitude; one test was conducted with a shut off from 5 m/s; and one test was conducted with a ramp from 3 m/s to 8 m/s.

**Figure 6:**
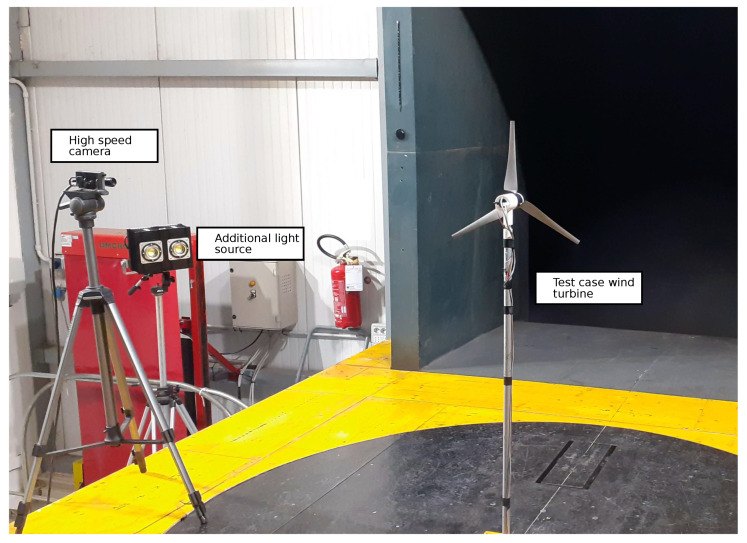
Experimental setup for wind tunnel tests.

As a small-scale wind turbine can rotate at much higher rotor speeds with respect to full-sized turbines, this test can be considered particularly stressful for the algorithm as video acquisition has to be captured at a high frame rate. For this reason, exploring the response to different air flows and speeds allowed us to find the limits of this method and its stability during transients. One of the most critical points is the maximum frame rate of the camera, which is strictly dependent on the acquisition parameters, such as the pixel dimensions of the frames and exposure. To achieve an adequate trade off between performance and image quality, we decided to acquire an array of 450×200 px with an exposure of 4 ms and a frame rate reaching 250 FPS. Figure 6 shows the arrangement of the equipment in the wind tunnel: the turbine was centered with respect to the inlet section of the flow, and the camera was installed on the left side of the chamber, pointing towards a blade of the rotor. Because of the low exposure time of the acquisition, an external LED light source was used in order to guarantee sufficient illumination.

Finally, some considerations need to be addressed regarding the optimal orientation of the camera with respect to the rotor. Even if an extensive sensitivity assessment regarding this aspect exceeds the purposes of the present study, it has to be taken in account that some preliminary tests were executed with different experimental setups, light conditions (laboratory or external environment) and turbine sizes in order to find the best location for the camera. The best results were obtained by placing the camera on one side of the turbine and pointing it tangentially towards a blade. The reasons for considering this arrangement as the most suitable are the fact that if the number of pixels undergoing a noticeable variation during a rotor revolution increases, the difference between the maximum and minimum values of the correlation increases; thus, the effect of noise or harmonics, which is discussed in detail in Section 4 and Section 5, decreases. Therefore, the general recommendation is to limit the amount of image occupied by non-rotating elements as much as possible in the background, tower or nacelle.

### 3.2. Field Measurement Campaign

Field measurement campaigns were performed in September 2019 at a wind farm sited in Italy comprising two 44 m rotor diameter turbines with 750 kW of power each. This test case can be considered peculiar, and thus worthy to be studied, because of its location and its long operation lifetime. Being situated at a mountain ridge, the wind turbines are often affected by icing and are in general subjected to a harsh environment consisting of strong gusts with non-negligible vertical components. According to this, and taking into consideration the fact that the wind farm has been operating since 1999, it is important to constantly survey these wind turbines with innovative, unintrusive methods without interfering with their energy production activity. In addition, the tested wind turbine model has a particular drive-train configuration with a variable speed gearbox with two alternating and fixed rotor speeds of 27 and 16 rotor RPM; this is therefore a useful property to test the video-tachometer algorithm.

An advantage of performing this kind of measurements with a full-scale wind turbine is the low rotational speed of the rotor with respect to that of the wind tunnel tests. This can be considered a facilitating element because the required frame rate from the camera decreases consistently to 25 RPM, allowing the exposure to be increased and for sufficiently bright pictures to be acquiredeven with weak solar light.

Critical issues to be noted in this context include the uncontrollable environmental variables that may affect the calculation of the correlation factor, such as the luminosity changes or the yaw movements of the wind turbine nacelle. As the correlation algorithm is based on the comparison of video frames with respect to a reference image, if some alteration in lighting or in nacelle position appears during the acquisition, the computed correlation would be compromised. For this reason, for the field test, the algorithm was upgraded in order to refresh the reference image as the mean value of correlation was subjected to drift.

**Figure 7:**
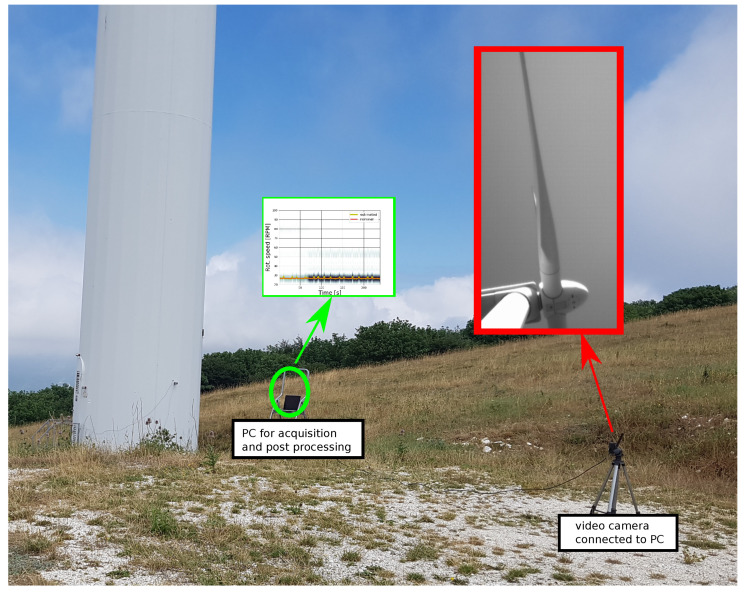
Instruments layout for in-site measurements.

Figure 7 shows how the equipment was installed at the base tower of the wind turbine to perform the measurement of the rotational speed. For this test, it was not possible to obtain a measured signal for an instantaneous rotational speed with the same sampling time as the estimates; in this sense, this application was particularly important because it compensated for the lack of information. From the wind turbine data sheets, it was possible to determine that the reference speed during the measurement campaign was 27 revolutions per minute, with possible oscillations due to wind gusts.

## 4. Results

**Figure 8:**
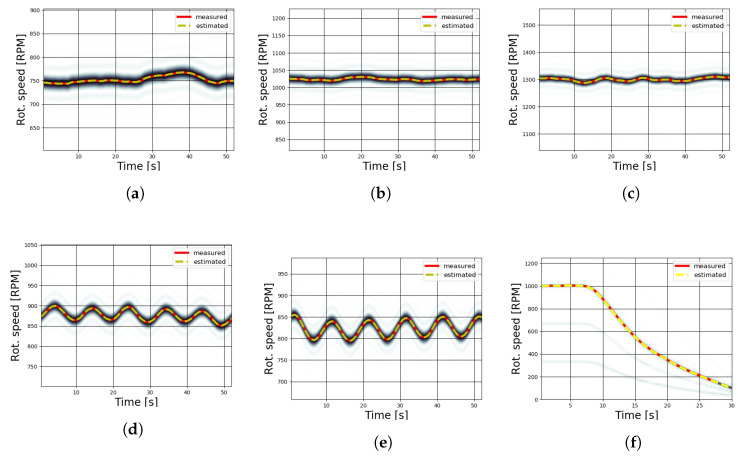
Estimated rotational speed from wind tunnel tests. (**a**) 4 m/s; (**b**) 5 m/s; (**c**) 6 m/s; (**d**) sinusoidal wind with a 4.5 m/s mean value and 0.3 m/s amplitude; (**e**) as the previous run, with 0.6 m/s of amplitude; (**f**) transitory from 5 m/s.

**Figure 9:**
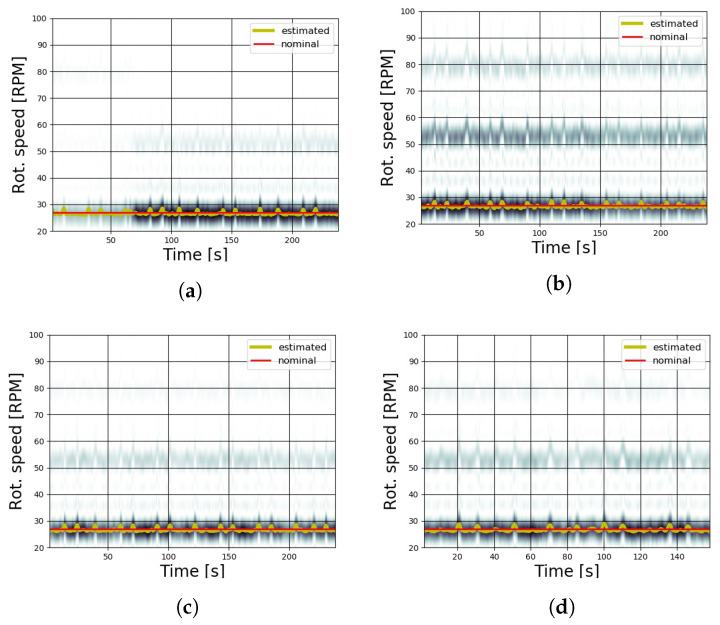
Spectrograms from four sample field measurements. The wind turbine has a constant nominal speed of 27 revolutions per minute.

The estimation of rotational speed for the wind tunnel tests was realized by processing the spectrograms of the virtual tachometer signals acquired from six different runs; the corresponding time–frequency plots are shown in Figure 8a–f. The accuracy of results from STFT usually depends on the choice of some parameters, such as the number of samples, which reflect the frequency and time resolution. For the following analysis, the spectrograms were realized using a Tukey window, with a 0.25 shape parameter, on 256 sample-length segments with an overlap of 200 samples. The effective resolution of the spectrogram was 0.977 Hz. By adding zero padding to the sample segment, it was possible to finely interpolate the signal in the frequency domain. At least 744 points of zero padding should be added to the 256 sample in order to reach a reliable measurement (i.e., an *RMSE* lower than 1.4 RPM). Increasing the length of the zero padding leads to a better estimation (lower *RMSE*) at the cost of higher computational effort. In Figure 8 and Figure 9, 9744 zero padding points have been used; an interval length greater than 256 samples leads to an insufficient time resolution.

The estimated rotational speed curves (shown by a dotted yellow line) were obtained by tracking the instantaneous maximum value of the spectrogram and applying the scale factor to calculate the shaft speed from the blade passing speed. The presence of harmonics is visible in most of the acquisitions, in particular in Figure 8f, even if their low intensity did not affect the algorithm, as the maximum value was always related to the rotational frequency.

Once the method was validated in the wind tunnel, the same approach was used in the field measurement campaign with the full-scale wind turbines, as described in Section 3.1. For this test, the rotor speed was nominally constant at 27 revolutions per minute, and four sessions were recorded (Figure 9a–d) in order to verify the functionality and the performance of the algorithm. The results show that the tracking of the spectrum maximum correctly estimated the rotor speed of the wind turbine, even in the case of the low variations caused by wind gusts. For some runs (as shown in Figure 9b,d), it appears that the spurious harmonics were more prominent with respect to the cases in the controlled laboratory environment. In these cases, the maximum values were also reached at the rotational frequency, and therefore the algorithm correctly estimated the rotational speed.

Having stated that the method of the correlation and tracking of spectrogram maxima can estimate the rotational speed of both small-scale wind turbines and full-scale turbines, even in different environmental conditions, the subsequent step is the discussion of the robustness of the methodology in order to determine the possible limits of the measurement system and its capability to track the spectrogram maxima. As has been noted above, the spectrograms of Figure 8 and Figure 9 show the presence of harmonics in addition to the rotational speed signal, which may cause the maximum tracking process to fail in the recognition of peaks if their intensity overtakes that of the rotational speed.

The robustness and the repeatability of the algorithm were investigated to ensure that the maximum values relative to the rotational speed had a much higher magnitude with respect to the background noise introduced by the harmonics. For this reason, in Table 1 and Table 2, for each test run, the mean value of the signal-to-noise ratio (SNR) is reported. The signal S is intended to be the maximum value of the spectrogram, relative to the tracked rotational speed, and the noise N is the mean value of STFT(T,f) (Equation (8)). This provides a metric to assess the predominance of the required signal with respect to noise. The results shown in Table 1 and Table 2 confirm that the signal related to the rotational speed is at least one order of magnitude larger than the mean value of the STFT both in wind tunnel and open-field tests. This is a crucial point, as the maximum tracking algorithm is therefore as efficient as the signal of the rotational speed is broad with respect to other undesired harmonics or noise. In the field measurements, the presence of spurious harmonics can be considered non-negligible: therefore, a necessary assessment has been conducted by calculating the mean difference between the peaks of the rotational speed and the first harmonic in order to ensure that the maximum tracking does not detect the rotational speed incorrectly if the first harmonic assumes values higher than the rotational speed. This check has been conducted only in consideration of the first harmonic, because the subsequent harmonics have lower maxima with respect to the first for all the conducted tests.

The percentage difference in Table 2 has been computed as

(9) $$diff_{\%}=\frac{peak_{rot}-peak_{harmI}}{peak_{rot}}\times 100$$

where $peak_{rot}$ is the peak value (maximum) of rotational speed in a time interval of the spectrogram, and $peak_{harmI}$ is the peak value (maximum) of the first harmonic in a time interval of the spectrogram.

**Figure 10:**
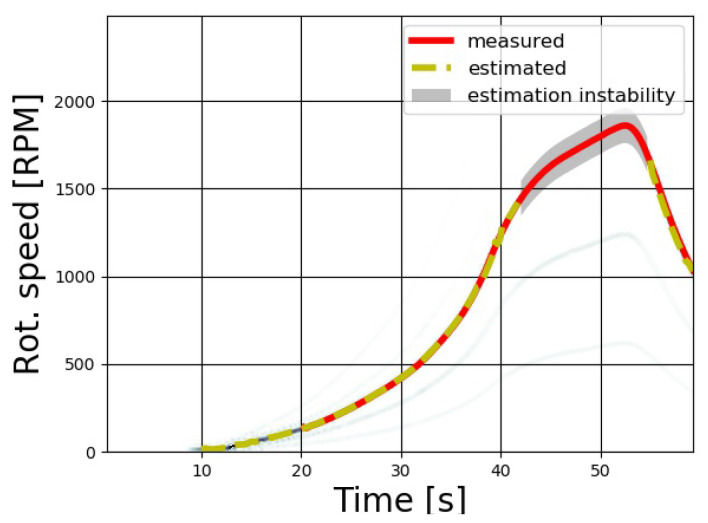
Wind ramp test at 0–8 m/s: the rotational speed quickly reaches 1900 RPM.

A stress test for the measurement equipment was performed in the wind tunnel facility, subjecting the test case wind turbine model to a sharp wind ramp from 0 m/s to 8 m/s and causing the rotational speed to reach up to 1900 RPM in about 50 s from the beginning of the run. As can be seen in Figure 10, the algorithm was able to follow the measured speed up to order of 1500 RPM with low error; beyond this limit, the estimated rotational signal began to be unstable as the maximum value of the spectrogram passed from the rotational speed to its first lower harmonic. This means that the operation of the maximum search does not guarantee that the highest value belongs to the required harmonic. As soon as the wind decreased and the speed slowed to under the level of 1500 RPM, the algorithm again correctly estimated the rotational speed. The impossibility of measuring rotor speeds above the order of 1500 RPM is due to the frame rate of the acquisition rather than the data analysis method.

Finally, an analysis has been conducted to determine the sensitivity of the presented algorithm to external factors concerning the noise that may affect the acquisition of images, especially in conditions of low illumination; i.e., when a low exposition time is required or when some meteorological variability appears in the open field.

**Figure 11:**
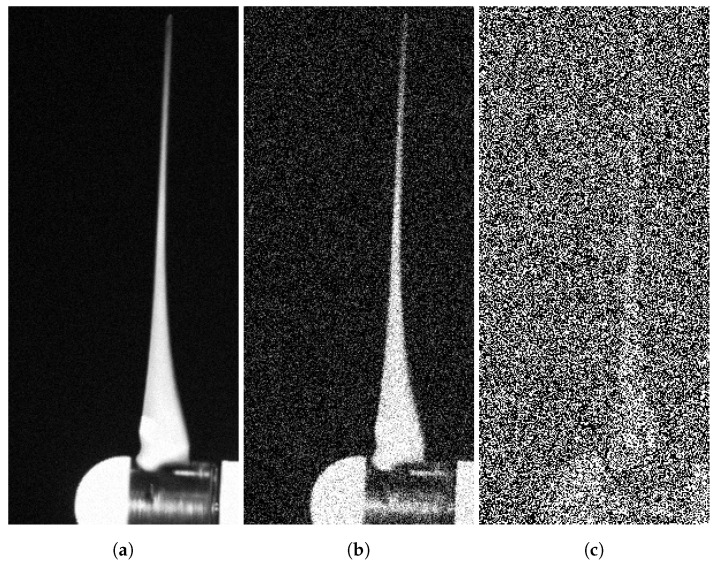
Different levels of white noise added to the video frames: (**a**) *σ*^2^ = 5; (**b**) *σ*^2^ = 50; (**c**) *σ*^2^ = 500.

To include random noise in this study, a Gaussian white noise signal was digitally added to the frames in one of the wind tunnel tests. A Gaussian distribution p(x) can be described by the following Equation (10):

(10) $$p\left(x\right)=\frac{1}{\sqrt{2\pi \sigma^{2}}}e^{-\frac{{\left(x-\mu \right)}^{2}}{2\sigma^{2}}}$$

where the mean value μ is equal to zero for the considered white noise and $\sigma^{2}$ is the variance. As a first test, one matrix with the same dimensions as the frames was generated with random values and then added to the reference image and to all the frames of the video acquisition. Three test values for the variance of the white noise were considered: $\sigma^{2}$ = 5, $\sigma^{2}$ = 50, $\sigma^{2}$ = 500 (Figure 11). This was done in order to determine the highest value admissible by the correlation algorithm. The effect of external noise was computed by using the root mean squared error (*RMSE*) between the time history of the measured and estimated rotational speed. In this situation, even in the case of the heaviest noise with $\sigma^{2}$ = 500, no noticeable deviations in the *RMSE* were found: its values were found to be 0.256 in all cases.

A second test was subsequently set up with the matrix of white noise, which was recalculated for each frame of the video; in this case, the random effect of the noise acted both in space and in time. The calculated *RMSE* valeus for the three cases were $RMSE_{5}=0.259,RMSE_{50}=0.255$ and $RMSE_{500}=17.136$. We can observe that the impact of noise becomes non-negligible when a random, time-varying white noise is added whose $\sigma^{2}$ becomes greater than 500. This can be considered to be a qualitative evaluation of the acceptable noise threshold for the algorithm to obtain reliable results.

## 5. Conclusions

In this study, a method to estimate the rotational speed of a wind turbine through contactless video acquisition has been presented. A straightforward motivation of this work is its possible application in the context of non-intrusive wind turbine condition monitoring techniques, but the potentiality of the proposed method is more general.

In the present study, a correlation-based algorithm has been proposed that employs a reference image of wind turbine blades and a series of frames extracted from a continuous recording while the machine is operating. Knowing the frame rate of the camera, the correlation coefficient with respect to the reference image is computed for each frame. As a result, a virtual tachometer signal is obtained that presents a periodicity corresponding to the blade passing frequency. Finally, the rotor speed time history is extracted by post-processing the virtual tachometer signal with STFT and tracking the maximum values.

The proposed method has been tested in a wind tunnel facility with a small-scale wind turbine model and then applied in a full-scale, industrial-size wind farm. The validation of the algorithm was set up in wind tunnel tests, because the wind turbine was equipped with an optical tachometer sensor, and therefore a comparison between the estimated rotational speed and the measured speed was possible. In the full-scale tests, no reference rotor speed signals were available, even if a reference nominal rotor speed was known from the wind turbine data sheet.

The response of the algorithm has been shown to be robust and repeatable even in consideration of the different conditions of the laboratory environment and the wind farm. On one hand (wind tunnel), there were conditions of controlled and constant lighting, and the critical points regarded the extremely high rotor speed reached by the test case wind turbine device (up to 1500 RPM) and its possible relevant variability in time in the case of transient wind or gusts. On the other hand (industrial wind farm tests), the nominal rotor speed was 27 RPM, and therefore there were no critical aspects as regards the management of the camera frame rate; in this case, the difficulties were constituted by the environmental variability in luminosity and by the dynamics of the rotor (such as yaw movements). In both cases, the speed estimation was considered to be reliable: the behavior of the signal-to-noise ratio shows that the effect of noise did not disturb the algorithm of maximum tracking as much as it compromised the quality of the rotor speed estimation. In fact, the harmonic required to estimate the rotational speed (the signal) was shown to be predominant, up to a factor of $10^{2}$, with respect to the mean value of the module in each STFT (the noise).

Further developments of the present study will involve the use of this methodology in combination with base tower vibration measurements (in a manner similar to [14]) for wind turbine drive-train condition monitoring applications in industrial wind farms. The approach proposed in this work could be particularly valid in the context of fault diagnosis for wind turbines which have not been equipped at their installation with condition monitoring systems because of economic considerations. These kinds of machines are at present typically reaching the end of their expected lifetime, and for this reason, they are typically operating out of warranties and full service agreements with the constructor company; therefore, condition monitoring for these wind turbines is mostly an overlooked topic, and the methodology proposed in this work might constitute a basis for setting up periodic inspections of these turbines.

## Figures and Tables

**Table 1:** Signal-to-noise ratio (SNR) evaluation for wind tunnel tests.

Run	S	N	SNR
4 m/s	$5.220\times 10^{-3}$	$5.742\times 10^{-5}$	$9.089\times 10^{1}$
5 m/s	$4.013\times 10^{-3}$	$3.831\times 10^{-5}$	$1.047\times 10^{2}$
6 m/s	$2.860\times 10^{-3}$	$2.935\times 10^{-5}$	$9.742\times 10^{1}$
Sinusoidal 0.3 m/s amplitude	$2.783\times 10^{-3}$	$3.098\times 10^{-5}$	$8.984\times 10^{1}$
Sinusoidal 0.6 m/s amplitude	$2.326\times 10^{-3}$	$2.772\times 10^{-5}$	$8.389\times 10^{1}$
Shut down	$1.892\times 10^{-3}$	$2.768\times 10^{-5}$	$6.209\times 10^{1}$

**Table 2:** SNR evaluation for in-field tests.

Run	S	N	S/N	% Difference (Mean)
1	$2.269\times 10^{-2}$	$2.234\times 10^{-4}$	$9.987\times 10^{1}$	$9.277\times 10^{1}$
2	$1.343\times 10^{-2}$	$2.172\times 10^{-4}$	$6.189\times 10^{1}$	$5.072\times 10^{1}$
3	$1.201\times 10^{-2}$	$1.364\times 10^{-4}$	$8.785\times 10^{1}$	$8.124\times 10^{1}$
4	$4.236\times 10^{-3}$	$5.176\times 10^{-5}$	$8.184\times 10^{1}$	$7.522\times 10^{1}$
